# Birth plan compliance and its relation to maternal and neonatal outcomes

**DOI:** 10.1590/1518-8345.2007.2953

**Published:** 2017-12-11

**Authors:** Pedro Hidalgo-Lopezosa, María Hidalgo-Maestre, Maria Aurora Rodríguez-Borrego

**Affiliations:** 1 PhD, Researcher, Instituto Maimónides de Investigación Biomédica de Córdoba (IMIBIC); 2 MSc, RN, Hospital San Juan de Dios, Córdoba, Espain; 3 PhD, Full Professor, Researcher, Instituto Maimónides de Investigación Biomédica de Córdoba (IMIBIC)

**Keywords:** Birth, Birth Plan, Newborn, Humanized Birth, Maternal and Child Health

## Abstract

**Objective:**

to know the degree of fulfillment of the requests that women reflect in their birth plans and to determine their influence on the main obstetric and neonatal outcomes.

**Method:**

retrospective, descriptive and analytical study with 178 women with birth plans in third-level hospital. Inclusion criteria: low risk gestation, cephalic presentation, single childbirth, delivered at term. Scheduled and urgent cesareans without labor were excluded. A descriptive and inferential analysis of the variables was performed.

**Results:**

the birth plan was mostly fulfilled in only 37% of the women. The group of women whose compliance was low (less than or equal to 50%) had a cesarean section rate of 18.8% and their children had worse outcomes in the Apgar test and umbilical cord pH; while in women with high compliance (75% or more), the percentage of cesareans fell to 6.1% and their children had better outcomes.

**Conclusion:**

birth plans have a low degree of compliance. The higher the compliance, the better is the maternal and neonatal outcomes. The birth plan can be an effective tool to achieve better outcomes for the mother and her child. Measures are needed to improve its compliance.

## Introduction

Women are increasingly involved in their own birthing processes. The birth plan is a tool that contributes to this fact, since it is a written document that the woman presents before delivery to the professionals who will assist her and reflect her preferences, expectations and fears about her own birthing process. The birth plan facilitates communication with professionals, improves women’s satisfaction, and promotes their participation and decision-making in their own birthing process^1^ ^-^ ^3^. This document is prepared by the woman and her partner during pregnancy and usually relies on the advice of the primary care midwifery^1^ ^-^ ^2^.

Just like the informed consent document, the birth plan is a supporting document to gather the user’s will. The free and informed decisions of the users must be respected, regardless of the form and title given to it, even if they are expressed only verbally^4^.

The use of birth plans is recommended by institutions such as the World Health Organization (WHO), as it encourages a more natural process of childbirth and avoids routine intervention procedures, such as early amniotomy, systematic episiotomy and others^5^.

In Spain, in the 1990s, several associations emerged against excessive intervention in childbirth care which, since the middle of the last century, has been institutionalized and subjected to an important degree of medicalization^6^. The first childbirth plans emerged in 2004 through some associations, but their use began to be extended after its institutionalization by the Ministry of Health, since the birth plan was included as a tool in the Normal Childbirth Strategy of the National Health System and in the corresponding Clinical Practice Guidelines on Childbirth, documents that collect scientific evidence and the best knowledge available^7^.

However, the use of childbirth plans is not exempt from controversy and may give rise to some degree of conflict with professionals, who may see diminished see their autonomy and professionalism^8^. Some authors point out that childbirth plans can create tensions between women and their caregivers, which can lead to negative attitudes that may negatively influence clinical care^9^ ^-^ ^11^. Among the main causes that are described as originating from this tension are the women’s dissatisfaction with the non-fulfillment of their expectations^2^ ^,^ ^12^.

On the other hand, there are some studies that found a lower rate of cesarean sections^13^ and better neonatal outcomes^14^ in women with birth plans, as compared to those who did not presented it, although these studies are very scarce.

In Andalusia, Spain, following the Humanization of Perinatal Care Project in Andalusia, the health system offers the possibility of presenting a childbirth plan^15^. There is a model of childbirth plan created by the institution itself, in which the woman has only to indicate the items that she considers appropriate, or she can make her own plan freely. The number of women currently presenting a birth plan is still low, though there is evidence of slowly increasing.

From the knowledge that many of the petitions that women reflected in their birth plans were not met, it was hypothesized that the greater the compliance, the better would be the outcomes after childbirth. The objective of this study was to know the degree of compliance of the proposals reflected in the birth plans and to determine their influence on the main obstetric (proportion of cesarean and vaginal delivery) and neonatal outcomes (Apgar test and umbilical cord arterial blood pH). 

## Method

This is a retrospective, cross-sectional, descriptive and analytical study carried out in third-level hospitals of the Public Health System of Andalusia. A third-level public hospital was selected in each province, the one with the highest coverage. The sample consisted of women who presented a birth plan at admission to the referral hospital between January 2009 and January 2013. Data were extracted anonymously from the corresponding medical records. The following inclusion criteria were considered: low risk gestation, cephalic presentation, single childbirth, delivered at term. Scheduled and urgent cesarean sections without labor were excluded. The sample size was determined using the Epidat software version 3.1. The final sample consisted of 178 clinical records of parturient women.

Sociodemographic and obstetric variables were analyzed to determine the characteristics of pregnant women and the degree of compliance with the birth plan. For this purpose, the birth plans were carefully examined and the four most requested preferences during the labor process were extracted, accounting each one with 25%. These four requirements were: avoiding use of oxytocin during labor, avoiding early amniotomy, freedom of movement during labor, therefore without continuous monitoring, and avoiding episiotomy. The variables that were evaluated were: proportion of cesareans and vaginal deliveries, Apgar test at 1 minute and 5 minutes, and umbilical cord arterial blood pH, considering pH < 7.20 as pathological.

A descriptive and inferential analysis of the variables was performed using the Statistic PASW software version 19. The qualitative variables were expressed in number (n) and percentages (%) and the quantitative variables expressed as mean and standard deviation (SD). We used hypothesis contrast statistical tests according to the type of variable. For bilateral contrasts, the chi-square test was used, with Fisher’s exact test in qualitative variables and Student’s *t*-test for quantitative variables. An error α of 5% (*p* ≤ 0.05) was assumed, showing the exact *p-*values for each statistical test.

The permission by the ethics committee of the different hospitals ensured the access and compilation of clinical records data. 

## Results

The final sample consisted of 178 women’s medical records. Regarding the general characteristics, it is worth noting that the mean age was 33.00 ± 4.32 years, with a minimum age of 19 and maximum of 42 years. The percentage of women with university degrees was 49.3%, of which 45% are from the health and education sector.

The obstetric outcomes showed that 75% of women were primigravidae, 73% had spontaneous onset of labor, while the remaining 27% had induced labor. Moreover, 43% of the women had an episiotomy, another 43% received oxytocin during delivery, 34% of the women underwent amniotomy, and 70% received epidural analgesia. Seventy-six percent of the women had continuous monitoring and the remaining 24% had intermittent monitoring, so they had freedom of movement. Among those monitored continuously, 68% were monitored externally, while 8% were monitored internally. The outcomes at the end of delivery were as follows: 67% of the women presented normal or spontaneous delivery, 19% had instrumented delivery, and 14% had a cesarean section. Table 1 details the sociodemographic and obstetric characteristics if the women.

Data on the fulfillment of the birth plan (Table 2) showed that 3.4% of the women had their birth plan not fulfilled in any of its points; for 27% of women, only 25% of their total preferences was met; for 32.5% of them the birth plan was met in 50%; for 29.2% of the women, it was fulfilled for the most part (75%); and the birth plan was fully met for only 7.9% of the women.

When comparing the degree of compliance of the birth plan with the outcomes obtained according to the type of delivery, it was observed that the percentage of vaginal deliveries increased as the fulfillment of the birth plan increased. Thus, when compliance was 50% or less, the proportion of vaginal deliveries was 81.3%, while when compliance was greater than or equal to 75%, vaginal deliveries reached 93.9%. Regarding the proportion of cesarean sections, when adherence was low (less than or equal to 50%), the percentage of cesarean deliveries was 18.8%; while when compliance was high (75% or higher), the percentage of cesarean sections dropped to 6.1%; *p*=0.023. Eighty-four percent of cesarean sections occurred in the group of women with 50% compliance or less, while in the group of women with a high degree of compliance, only 16% of cesareans occurred. The data are shown in Table 3.

**Table 1:** Characteristics of women who presented a birth plan (N = 178). Third level hospitals of the Public Health System of Andalusia, ACS, Spain, 2009-2013

**Variable**	**n**	**(%)**
**Study level**		
**Primary**	**35**	**(23.3)**
**Secondary**	**41**	**(27.3)**
**University**	**74**	**(49.4)**
**Pregnancy**		
**Primigravidae**	**134**	**(75.3)**
**Secundigravidae**	**37**	**(20.8)**
**Tertigravidae**	**7**	**(3.9)**
**Gestational week**		
**< 40**	**66**	**(37.0)**
**40 - 40+6**	**64**	**(36.0)**
**> 41**	**48**	**(27.0)**
**Onset of labor**		
**Spontaneous**	**130**	**(73.0)**
**Induced**	**48**	**(27.0)**
**Previous cesarean section**	**8**	**(4.5)**
**Epidural anesthesia**	**124**	**(69.7)**
**Early amniotomy**	**61**	**(34.3)**
**Episiotomy**	**76**	**(43.0)**
**Use of oxytocin**	**75**	**(42.1)**
**Monitoring**		
**Intermittent**	**42**	**(24.0)**
**External continuous**	**120**	**(68.0)**
**Internal**	**16**	**(8.0)**
**Type of delivery**		
**Normal**	**119**	**(66.9)**
**Instrumental**	**34**	**(19.1)**
**Cesarean section**	**25**	**(14.0)**

**Table 2:** Conformity with the birth plan. Third level hospitals of the Public Health System of Andalusia, ACS, Spain, 2009-2013

**Compliance with the birth plan (%)**	**n**	**(%)**
**0**	**6**	**(3.4)**
**25**	**48**	**(27.0)**
**50**	**58**	**(32.5)**
**75**	**52**	**(29.2)**
**100**	**14**	**(7.9)**

**Table 3:** Maternal and neonatal outcomes according to degree of compliance of the birth plan. Third level hospitals of the Public Health System of Andalusia, ACS, Spain, 2009-2013

**Variable**	**Compliance** **≤50%**		**Compliance** **≥75%**		**p**
	**n**	**%**	**n**	**%**	
Type of delivery*					**0.023**
**Vaginal**	**91**	**(81.3)**	**62**	**(93.9)**	
**Cesarean section**	**21**	**(18.8)**	**4**	**(6.1)**	
**umbilical cord pH** ^**†**^					
**pH ≥ 7,20**	**76**	**(85.4)**	**48**	**(98.0)**	**0.024**
**pH < 7,20**	**13**	**(14.6)**	**1**	**(2.0)**	
Apgar 5min*					
**> 7**	**108**	**(96.4)**	**65**	**(98.5)**	**0.653**
**≤7**	**4**	**(3.6)**	**1**	**(1.5)**	
**Apgar 1min** ^**†**^					
**> 7**	**95**	**(88.8)**	**57**	**(98.3)**	**0.034**
**≤7**	**12**	**(11.2)**	**1**	**(1.7)**	

*N=178, †N=138, †N=165

Data obtained with the chi square statistical test and Fisher’s exact test

*p*-Value adjusted according to Finner’s method

Regarding neonatal outcomes (Table 3), children from mothers with high-compliance birth plans scored higher on 1 min Apgar test and had better scores on umbilical cord pH than children from mothers with low birth plan compliance. Thus, the low compliance group showed a pH <7.20 rate of 14.6%, which is much higher than the high compliance group, which was only 2% (*p*=0.024). There were no significant differences in the 5 min Apgar test. 

## Discussion

Analyzing the results on the degree of fulfillment of the birth plan among the women who presented it, the majority (63%) had the plan fulfilled or performed with mostly 50% of their requests, moreover, only 37% of the women had their birth plans fulfilled for the most part, and of these, only 8% had their plans fully fulfilled. It is logical to think that postpartum satisfaction will be directly proportional to the degree of fulfillment of the expectations met and fulfilled, as many authors suggest^3^ ^,^ ^12^ ^-^ ^13^ ^,^ ^16^.

The reasons for this low degree of compliance may be multiple, but it is necessary to mention here two main ones. First of all, as already mentioned, the course of the labor process is uncertain and can be complicated at any time, or unforeseen events may arise which may lead to a breach of the birth plan requirements and a change in the course of events. On the other hand, a certain tension can be generated between the parturient and the professional who assists her, due to the non-acceptance of the loss of professional autonomy; in these cases, the birth plan may act more likely as a barrier^8^ ^-^ ^11^. As a way to reduce this tension, the dialogue between the parties and the importance of prenatal education are mentioned^8^ ^,^ ^17^. Certain authors state that women who presented a birth plan are less satisfied with their midwives than women in the control group^6^. However, in a study conducted in a hospital in Spain, the authors found that women were satisfied with their midwives and 95% would resubmit a birth plan, although only 43% stated that their expectations were met^18^. Some authors did not find significant differences in satisfaction between the two groups^19^, while others found greater satisfaction in women with birth plans^20^. 

Regarding the results found, it is noteworthy that 84% of cesarean sections occurred in the group of women with adherence to plan less than or equal to 50%, whereas in the group with a high degree of compliance, 75% or more, only 16% of all cesarean sections were performed. In this sense, although no studies have been found that directly correlate the fulfillment of birth plans with the outcomes, there are some studies that concluded that women with birth plans had a lower risk of cesarean sections^20^ ^-^ ^22^. Other authors suggest that women with birth plans do not have a higher cesarean rate than those without a birth plan^23^. Moreover, other studies show that there were no significant differences in the cesarean rate between the two groups^14^ ^,^ ^24^.

The neonatal outcomes of the present study show that the higher the compliance of the birth plan, the better the Apgar score and the umbilical cord pH range. Other studies do not show any differences in the Apgar test between newborns of mothers with or without a birth plan^10^ ^,^ ^14^, except for one study in which a better average Apgar score was found in children born to women with a birth plan^20^. As for the outcomes on the variable pH of the umbilical cord arterial blood, it is even more difficult to compare them with those of other studies, because no similar study was found. However, in an earlier study by this research team on birth plan in which the degree of compliance was not taken into account, the offspring of mothers with birth plans had a lower proportion of pathological pH than the children of mothers of a control group^14^. Certain parallelism could be established with studies on birth centers or alternative centers where low intervention deliveries occur. In this sense, there are authors who compared measurements of cord pH and found that the mean value was higher in children born in birth centers than those who were born conventionally in a hospital^25^. However, presenting a birth plan does not only mean having a childbirth with fewer interventions; it should also be taken into account that these women tend to be more prepared, more controlled and have a greater participation in the process, etc., elements that may play an important role in reducing the degree of anxiety and stress during the delivery process.

The limitations of this study were mainly the heterogeneity of the caregiver professionals, as well as the heterogeneity of the birth plans themselves, since in some cases the institutionalized model is presented and in others, an original manuscript by the woman herself.

## Conclusion

In conclusion, the birth plan has a low degree of compliance, since only 37% of the women who presented it were mostly fulfilled. However, there is a direct relationship between obtaining a higher degree of compliance of the birth plan by obtaining better outcomes for both the mother and her child. Thus, as compliance with the birth plan increases, the cesarean rate decreases and the outcomes in the one minute Apgar test and the umbilical cord pH improve.

Implications for clinical practice: the birth plan can be an effective tool to favor a more natural and physiological delivery process, better communication with professionals, greater control of the labor process, better obstetric and neonatal outcomes and greater satisfaction. Improving the degree of fulfillment of the birth plans is key in obtaining these outcomes. Policies are needed to encourage the use of birth plans and to improve its implementation and compliance.
